# A multi-center effectiveness comparison study of fruquintinib with constructed external control cohort of other targeted kinase inhibitors using real-world data in third-line treatment of metastatic colorectal cancer

**DOI:** 10.3389/fonc.2022.1044328

**Published:** 2022-11-24

**Authors:** Ying Jin, Jin Li, Lin Shen, Jianming Xu, Yanqiao Zhang, Jingdong Zhang, Hongming Pan, Xiujuan Qu, Yamin Chen, Qiang Zhang, Jinnan Li, Miaomiao Sun, Shukui Qin

**Affiliations:** ^1^ Department of Medical Oncology, Sun Yat-Sen University Cancer Centre, Guangzhou, China; ^2^ Department of Medical Oncology, Shanghai Oriental Hospital Affiliated Tongji University East Hospital, Shanghai, China; ^3^ Department of Gastrointestinal Oncology, Beijing Cancer Hospital, Beijing, China; ^4^ Department of Medical Oncology, The Fifth Medical Centre of Chinese PLA General Hospital, Beijing, China; ^5^ Department of Gastroenterology, Harbin Medical University Cancer Hospital, Harbin, China; ^6^ Department of Gastroenterology, Liaoning Cancer Hospital and Institute, Shenyang, China; ^7^ Department of Medical Oncology, Sir Run Run Shaw Hospital, College of Medicine, Zhejiang University, Hangzhou, China; ^8^ Department of Medical Oncology, The First Hospital of China Medical University, Shenyang, China; ^9^ Department of Medical Oncology, First Affiliated Hospital of Dalian Medical University, Dalian, China; ^10^ Eli Lilly and Company, Lilly China Drug Development and Medical Affairs Centre, Shanghai, China; ^11^ Department of Medical Oncology, People’s Liberation Army (PLA) Cancer Centre of Jinling Hospital, Nanjing, China

**Keywords:** fruquintinib, FRESCO, real-world data, metastatic colorectal cancer, tyrosine kinase inhibitors, progression free survival

## Abstract

**Objective:**

The objective of this study was to assess the comparative efficacy in third-line setting for metastatic CRC (mCRC) patients using matched population of FRESCO trial with fruquintinib and real-world data with other TKIs.

**Materials and methods:**

The arm of fruquintinib from the FRESCO phase III trial (NCT02314819) included the data of patients with metastatic CRC that progressed after at least two lines of chemotherapy and received fruquintinib treatment. An external control arm was constructed using real-world data (RWD) of patients who received other TKIs based on key eligibility criteria of FRESCO. The baseline characteristics of two arms was balanced by propensity score matching (PSM). The Kaplan–Meier method and Cox proportional hazard model was used to evaluate progression free survival (PFS) and to estimate hazard ratios (HRs) and 95% confidence intervals (CIs), respectively.

**Results:**

Overall, 128 patients were successfully matched by PSM in each, fruquintinib and other TKIs group. The patients in fruquintinib group showed significant increase in median PFS than other TKIs (3.71 *vs.* 2.49 months, HR = 0.67, 95%CI, 0.48-0.94, *p =* 0.019). In the subgroup analysis, fruquintinib showed a significant benefit in PFS compared with other TKIs among patients undergoing two or three previous chemotherapy regimens (HR 0.58, 95%CI 0.40-0.84; *p*=0.004), with rectum as primary disease site (HR 0.52, 95%CI 0.31-0.87; *p*=0.013), with left sided primary tumor location (HR 0.62, 95%CI 0.42-0.90; *p*=0.011), with multiple metastasis sites (HR 0.68, 95%CI 0.48-0.97; *p*=0.034) and with lung metastasis (HR 0.65, 95%CI 0.43-0.98; *p*=0.042).

**Conclusion:**

With the approach of establishing the external control arm from RWD, this study has demonstrated that treatment with fruquintinib significantly prolonged PFS as compared to other TKIs in patients as third-line mCRC treatment.

## 1 Introduction

Colorectal cancer is the third most common cause of cancer and second leading cause of mortality in the world (1). Metastatic colorectal cancer (mCRC) itself has a high disease burden in China, with the number of new incident cases increased from 0.37 to 0.55 million with 0.29 million death cases, which accounts for around 8% of all cancer deaths in 2019 (2). Approximately, 20% patients identify as mCRC at their first diagnosis and 50% will develop eventually into mCRC (3). Patients with mCRC have poor prognosis, and hence are difficult to treat (4). It has been observed that four in every five mCRC tumors are unresectable (4) with a five-year survival rate of only 0.9% without any treatment (4).

According to the Chinese Society of Clinical Oncology (CSCO), chemotherapy is standard treatment option for patients with unresectable mCRC to prolong their survival and to improve the quality of life (4, 5). CSCO recommends chemotherapeutic agents such as FOLFOX, FOLFIRI, XELOX in combination with epidermal growth factor receptor inhibitors (EGFRi) or vascular endothelial growth factor inhibitors (VEGFi) as first-or second-line treatment for patients with mCRC (4–6). The previous clinical trials have reported significant improvement in the progression-free survival (PFS) and overall survival (OS) of patients with mCRC when treated with targeted therapy and chemotherapy as compared to chemotherapy alone (4–6).

Despite the promising response of these first-line and second-line therapy, patients still experience disease progression (7). In this context, the National Comprehensive Cancer Network (NCCN) and CSCO guidelines recommended tyrosine kinase inhibitors (TKI) drugs as third-line treatment option for patients with mCRC (8). The commonly used TKIs for patients with mCRC as a third-line treatment in Chinese clinical practice include fruquintinib, regorafenib (8), apatinib and anlotinib (8).

Fruquintinib, a highly selective small-molecule TKI, has demonstrated its efficacy in treating patients with mCRC (8, 9). In a previous randomized, multicenter, phase 3, FRESCO trial (NCT02314819), fruquintinib showed significant survival benefit (PFS & OS) as a third-line treatment in Chinese patients with mCRC who progressed after standard second-line therapy. Similarly, a real-world retrospective study in Chinese patients with mCRC revealed comparable survival to the FRESCO study **(** 10 **).** Moreover, subgroup analysis revealed clinically meaningful improvement in OS (11), objective response rate (11), and disease control rate (11) with fruquintinib in both prior and non-prior targeted therapy.

Apart from these above-mentioned studies, there are no head-to-head trials comparing the efficacy of various TKIs especially in third-line setting for patients with mCRC. Currently, published results considering the efficacy between various TKIs are controversial. The most recently published observational study with 366 patients by Zhang et al, 2022 demonstrated that regorafenib and fruquintinib had similar efficacy, and a longer OS for regorafenib was observed in the sequence analysis but needs to be further validated (12). Several network meta-analyses have also analyzed the differences of the efficacies between fruquintinib and other TKI (e.g. regorafenib) (13–15). A recent network meta-analysis of eight randomized control trials (RCTs) in patients who progressed beyond the second-line settings demonstrated the ability of fruquintinib in improving PFS especially in patients with wild-type KRAS mCRC (16). This trend for PFS was also observed in the other meta-analysis but no significant difference was observed in OS (14).Another network meta-analysis revealed fruquintinib and regorafenib had similar effect on PFS (15) while an indirect comparison demonstrated a lower PFS for regorafenib than fruquintinib for mCRC beyond second-line therapy (17).

These meta-analyses were however limited by the small number of included RCTs and higher heterogeneity due to baseline characteristics among enrolled populations. In addition, since these studies were conducted at the study level, it might not present the confounding variables that would be present at the patient level (9, 13, 14, 16). Thus, efficacy evaluation based on a more comparable population is required. To address all these research gaps, the aim of this study was to assess the comparative efficacy in third-line setting for patients with mCRC using matched population of FRESCO trial with fruquintinib and real-world data (RWD) with other TKIs.

## 2 Materials and methods

### 2.1 Study design

The FRESCO phase III trial consisted of the patients who had metastatic CRC that progressed after at least two lines of chemotherapy and received either fruquintinib or placebo (11). The data of patients receiving fruquintinib were included for this study. An external control arm was constructed using RWD derived from hospital information system (HIS) database of six top-tier hospital in China. The patients diagnosed with mCRC between Jan 1, 2015 and Feb 28, 2018 and who have received other TKIs (e.g., regorafenib, apatinib, anlotinib) were included in this group. To select matched population for comparison, the key eligible criteria of real-world population were mirrored with that of FRESCO trial as much as possible, which is as follows.

### 2.2 Inclusion criteria

a. Patients aged between 18-75 years
b. The patients were diagnosed with metastatic colorectal adenocarcinoma (stage IV) which was confirmed by histology and/or cytology.
c. The patients should have received at least second-line standard chemotherapy (must include fluorouracil, oxaliplatin and irinotecan) but failed to respond to prior treatments and progressed to disease.

### 2.3 Exclusion criteria

a. The patients who have participated in clinical trial of other drugs in the past 4 weeks.
b. The patients who have used prior VEGFR inhibitors.
c. The patients who were having central nervous system metastasis or previous brain metastasis were excluded.
d. The patients with clinically detectable second primary malignancy at index date or history of other malignancies in the past 5 years (except for fully-treated skin basal cell carcinoma or cervical carcinoma in situ) were excluded.

This is a study with secondary use of data. At the time of study inclusion, the data had been deidentified to protect subject privacy and hence the study Consent to Release Information has been exempted.

### 2.4 Study endpoints

The primary efficacy endpoint of the study was PFS which was defined as time from randomization (for FRESCO trial patients) or time of the first use of non-fruquintinib TKI date of reviewed medical records (for other TKIs patients from RWD) to disease progression or death (Detailed progression/censoring rules are available in **Appendix 1**).

### 2.5 Statistical analysis

Descriptive statistics were used to summarize the baseline characteristics. To balance the baseline cohort characteristics of FRESCO trial with RWD and reduce selection bias propensity score-matching (PSM) was performed. The PSM was carried out with a caliper (0.2 standard deviations) and matched 1:1 greedy propensity score between fruquintinib and other TKIs groups. Gender, age, previous chemotherapy regimens, prior use of VEGFi, prior use of EGFRi, presence of multiple metastasis, presence of liver metastasis and primary site of CRC were included in the PSM model. Further, the Kaplan–Meier method was used to evaluate PFS, censoring the rules of real-world patients are consistent with FRESCO (**Appendix 1**). A Cox proportional hazard model was used to estimate hazard ratios (HRs) and 95% confidence intervals (CIs). A subgroup analysis was also carried out which included analysis of variables including time from first diagnosis to baseline (≤18 months/> 18 months), previous chemotherapy regimens (2 or 3/> 3), prior use of VEGFi or/and EGFRi (Yes/No), primary disease site (Colon/Rectum), primary tumor location (Left/Right), metastasis (Single/Multiple), lung metastasis (Yes/No) and liver metastasis (Yes/No). All tests were two-sided, and *p* < 0.05 was considered statistically significant. Data was analyzed using SAS version 9.4 (*SAS Institute Inc.*) and R (*The R Foundation*).

## 3 Results

### 3.1 Patient’s baseline characteristics

The study included data of 278 patients from the FRESCO trial and 257 patients from real-world. **Table 1** summarizes the baseline characteristics for the two groups. The other TKIs group consisted of elder patients than fruquintinib group (mean age 57.35 *vs*. 54.33 years) while the time from diagnosis to first use of either TKIs or fruquintinib was found out to be similar in ≤18 and >18 months. Mostly, the patients of two groups diagnosed at time of first diagnosis were at stage IV, and the proportion is larger in other TKIs group (55.6%) than that in fruquintinib group (42.1%). Both the cohorts were similar with respect to primary tumor location (left) and most common disease site (colon). The major metastasis site was liver with 73.5% in other TKI group and lung with 67.6% in fruquintinib group. Majority of the patients were having multiple metastasis with 68.9% in other TKIs groups, compared to those with 95.3% in fruquintinib group. Approximately, 31.7% patients in fruquintinib group have undergone more than three regimens of chemotherapy previously, while only 11.3% in other TKIs group had undergone more than three regimens previously. Also, 60.1% patients in fruquintinib group had never used any targeted treatment in previous chemotherapy; however, the other TKIs group had only 26.8% patients without any targeted therapy.

**Table 1:** Baseline characteristic of FRESCO compared to other TKIs group from real-world data before and after matching.

		Before PSM			After PSM			Statistical test applied
Variables		Other TKIs N (%)	Fruquintinib N (%)	*p*	Other TKIs N (%)	Fruquintinib N (%)	*p*	
**N**		257	278		128	128		
**Age (mean (SD))**		57.35 (10.24)	54.33 (10.70)	0.001	56.15 (10.33)	56.09 (10.94)	0.967	t-test
**Age group (%)**	<65 years	188 (73.2)	228 (82.0)	0.014	99 (77.3)	96 (75.0)	0.66	Pearson Chi-square test
	≥65 years	69 (26.8)	50 (18.0)		29 (22.7)	32 (25.0)		
**Sex (%)**	F	97 (37.7)	120 (43.2)	0.202	60 (46.9)	50 (39.1)	0.207	Pearson Chi-square test
	M	160 (62.3)	158 (56.8)		68 (53.1)	78 (60.9)		
Time from first diagnosis to randomization (median [IQR])^*^		20.59	21.40		26	18.4		Wilcoxon rank-sum test
		[13.28, 32.95]	[14.20, 35.10]	0.456	[15.40, 38.42]	[13.40, 28.85]	0.039	
**Time from first diagnosis to randomization group (%)**	≤18 months	109 (42.4)	116 (41.7)	0.624	43 (33.6)	62 (48.4)	0.016	Pearson Chi-square test
	>18 months	148 (57.6)	161 (57.9)		85 (66.4)	66 (51.6)		
**Colorectal cancer stage at first diagnosis (%)**	Stage I	2(0.8)	8 (2.9)		1 (0.8)	2 (1.6)		Pearson Chi-square test
	Stage II	23 (8.9)	34 (12.2)		17 (13.3)	12 (9.4)		
	Stage III	58 (22.6)	118 (42.4)	<0.001	36 (28.1)	51 (39.8)	0.005	
	Stage IV	143 (55.6)	117 (42.1)		60 (46.9)	62 (48.4)		
	Missing	31 (12.1)	1 (0.4)		14 (10.9)	1 (0.8)		
Primary tumor location (%)^*^	Left	201 (78.2)	214 (77.0)	0.545	102 (79.7)	101 (78.9)	0.877	Pearson Chi-square test
	Right	53 (20.6)	56 (20.1)		26 (20.3)	27 (21.1)		
Primary disease site (%)^*^	Colon	153 (59.5)	147 (52.9)		70 (54.7)	64 (50.0)		Pearson Chi-square test
	Rectal	102 (39.7)	125 (45.0)	0.163	58 (45.3)	60 (46.9)	0.116	
	Colon-rectal	2 (0.8)	6 (2.2)		0 (0.0)	4 (3.1)		
Liver metastasis (%)^*^	Yes	189 (73.5)	185 (66.5)	0.078	85 (66.4)	97 (75.8)	0.098	Pearson Chi-square test
Lung metastasis (%)^*^	Yes	129 (50.2)	188 (67.6)	<0.001	75 (58.6)	90 (70.3)	0.05	Pearson Chi-square test
**Metastatic (%)**	Single	80 (31.1)	13 (4.7)	<0.001	22 (17.2)	12 (9.4)	0.066	Pearson Chi-square test
	Multiple	177 (68.9)	265 (95.3)		106 (82.8)	116 (90.6)		
**Prior surgery (%)**	Yes	154 (59.9)	264 (95.0)	<0.001	84 (65.6)	118 (92.2)	<0.001	Pearson Chi-square test
**Prior Radiation (%)**	Yes	47 (18.3)	85 (30.6)	0.001	32 (25.0)	29 (22.7)	0.66	Pearson Chi-square test
Previous chemotherapy regimens (%)^*^	2 or 3	228 (88.7)	190 (68.3)	<0.001	102 (79.7)	106 (82.8)	0.522	
	>3	29 (11.3)	88 (31.7)		26 (20.3)	22 (17.2)		Pearson Chi-square test
**Chemotherapy and pharmacological treatment (%)**	Yes	257 (100.0)	278 (100.0)	NA	128 (100.0)	128 (100.0)	NA	Pearson Chi-square test
**Prior use of VEGF inhibitors** (%)^*^	Yes	154 (59.9)	84 (30.2)	<0.001	54 (42.2)	57 (44.5)	0.705	Pearson Chi-square test
**Prior use of EGFR inhibitors** (%)^*^	Yes	71 (27.6)	40 (14.4)	<0.001	30 (23.4)	23 (18.0)	0.28	Pearson Chi-square test
	Neither	69 (26.8)	167 (60.1)		55 (43.0)	57 (44.5)		
**Prior chemotherapy with VEGF and EFGR inhibitors (%)**	VEGF only	117 (45.5)	71 (25.5)	<0.001	43 (33.6)	48 (37.5)	0.737	Pearson Chi-square test
	EGFR only	34 (13.2)	27 (9.7)		19 (14.8)	14 (10.9)		
	Both	37 (14.4)	13 (4.7)		11 (8.6)	9 (7.0)		

CI, confidence interval; EGFR, epidermal growth factor receptor; F, females; M, males; N, number of patients; PSM, propensity score-matching; SD, standard deviation; TKI, tyrosine kinase inhibitor; VEGF, vascular endothelial growth factor.

* were the covariates that were used for propensity score matching.

NA, not applicable.

PSM was performed to balance the baseline patient characteristics of these two cohorts. Patients with missing baseline variables included in PSM model was removed. After PSM, 128 patients were included in each fruquintinib and other TKIs group. In other TKIs group, 55% of patients were treated with regorafenib, 43% of patients with apatinib while the remaining 2% of patients were treated with anlotinib. There was no statistically significant difference between the two groups with respect to age, gender, primary tumor location, primary site of disease, lung metastasis, liver metastasis, metastatic sites, prior radiation therapy, previous chemotherapy regimens and prior chemotherapy with VEGFi and EFGRi (**Table 1**). The time from first diagnosis to first use of TKIs was shorter for non-fruquintinib TKI users (33.6% in ≤18 months group) than that for fruquintinib users (48.4% in ≤18 months group). A statistically significant larger proportion of patients who had undergone prior surgery was observed in fruquintinib group (92.2%) than that in other TKI group (65.6%).

### 3.2 Efficacy outcomes

#### 3.2.1 Progression-free survival

**Figure 1:**
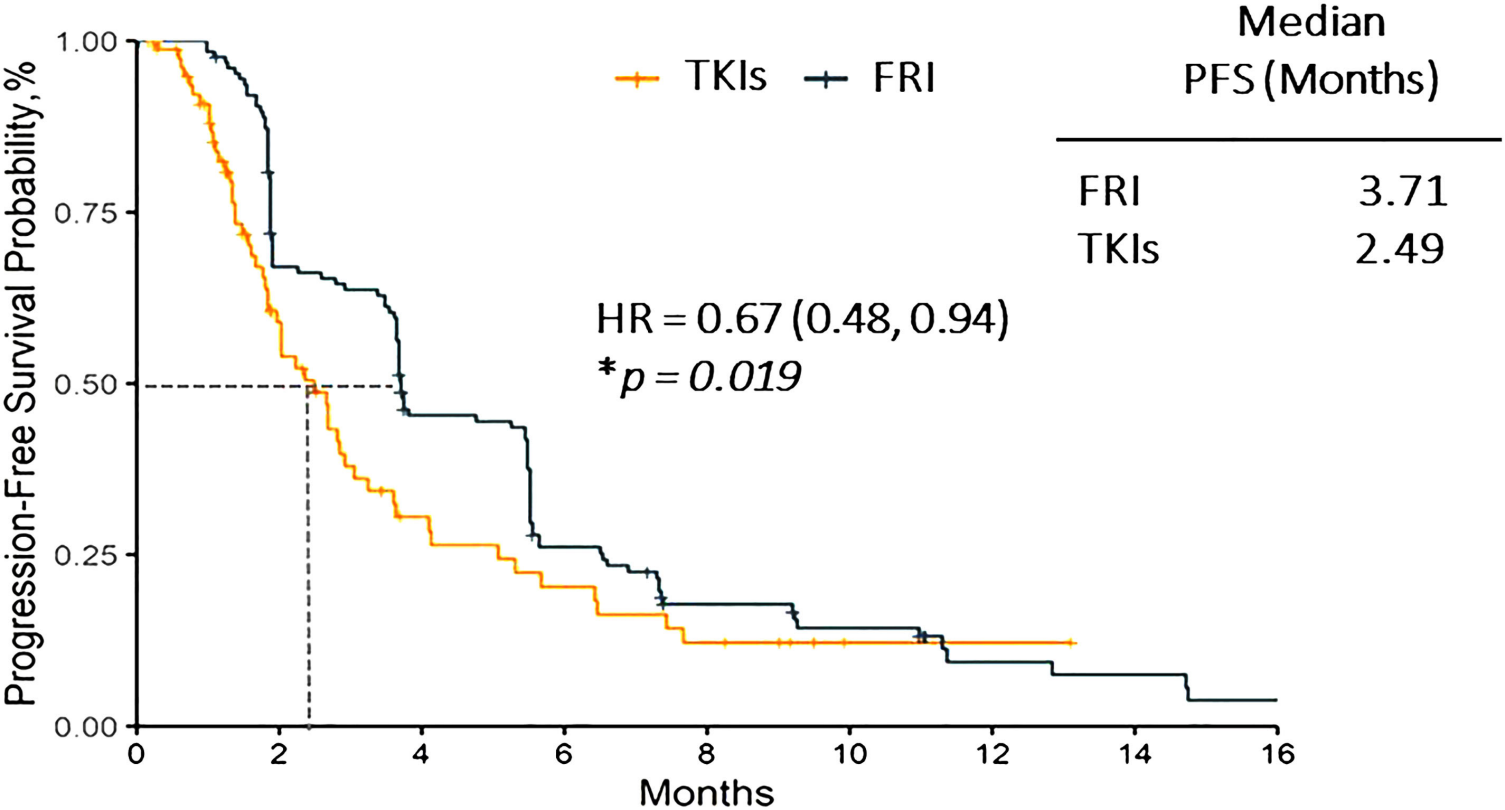
Progression free survival of fruquintinib *vs.* other TKIs group. FRI, Fruquintinib; HR, Hazard ratio; PFS, progression free survival; TKI, tyrosine kinase inhibitor. * P-value in Cox regression model. *p* < 0.05 considered statistically significant.

After matching, the median PFS was 3.71 months (95%CI, 3.65-5.49) in frquintinib group and 2.49 months (95%CI, 1.84-3.25) in other TKIs group. The estimated HR between these two groups was 0.67 (95%CI, 0.48-0.94, *p=*0.019) (**Figure 1**).

#### 3.2.2 Subgroup analysis for PFS between two groups

**Figure 2:**
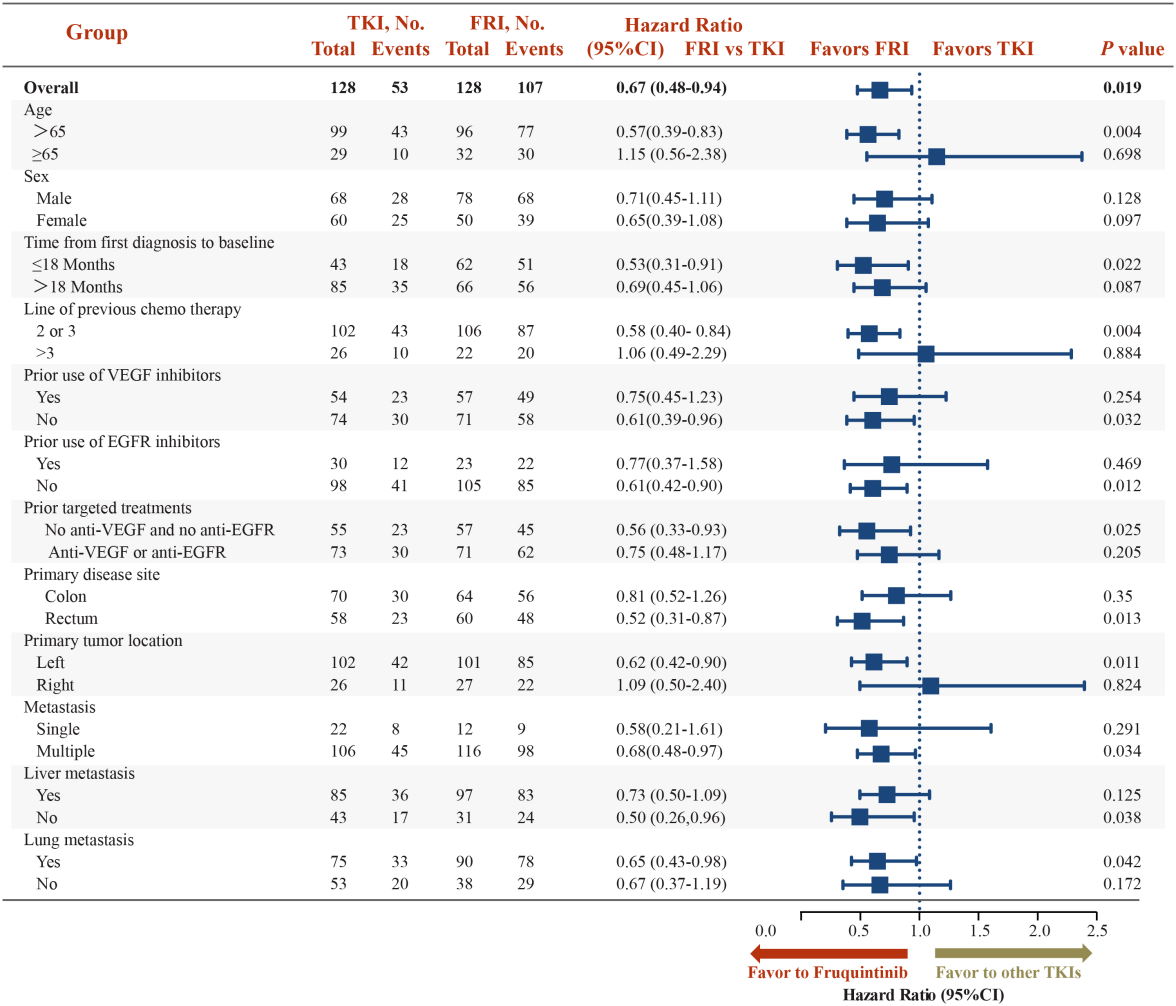
Subgroup Analysis: progression free survival of fruquintinib *vs.* other TKIs group. CI, confidence interval; EGFR, epidermal growth factor receptor; FRI, Fruquintinib; TKI, tyrosine kinase inhibitor; VEGF, vascular endothelial growth factor. P-value in Cox regression model. *p < 0.05 considered statistically significant.

The subgroup analysis revealed significant difference between fruquintinib and other TKIs groups among patients taking two or three previous chemotherapy regimens (HR 0.58, 95%CI 0.40-0.84; *p*=0.004) with rectum as primary disease site (HR 0.52, 95%CI 0.31-0.87; *p*=0.013), with left sided primary tumor location (HR 0.62, 95%CI 0.42-0.90; *p*=0.011), at multiple metastasis site (HR 0.68, 95%CI 0.48-0.97; *p*=0.034) and with lung metastasis (HR 0.65, 95%CI 0.43-0.98; *p*=0.042) (**Figure 2**).

## 4 Discussion

This study compared the patients from the FRESCO phase III trial who had failed standard second-line treatment for mCRC and received fruquintinib as a third-line treatment with the patients from RWD who received other TKIs such as regorafenib, apatinib and anlotinib. Our results indicated that fruquintinib had significantly prolonged the median PFS as compared to other TKIs for third-line mCRC treatment. The median PFS of fruquintinib arm in our study was found to be 3.71 months, which is consistent with FRESCO study (3.7 months for fruquintinib group) (11).

A most recent disclosure of phase III, multiregional clinical trial, FRESCO-2 trial (NCT0432253) in patients with refractory mCRC for more than third-line treatment, had showed that fruquintinib not only significantly improved OS (median: 7.4 m *vs.* 4.8 m; HR=0.66; [95% CI: 0.55, 0.80]; p<0.001) but also PFS (median: 3.7 m *vs.* 1.8 m; HR=0.32; [95% CI: 0.27, 0.39]; p<0.001) as compared to placebo. This evidence signifies the clinical benefit of fruquintinib even for mCRC treatment beyond third-line (18).

In other real-world studies, the treatment with fruquintinib has even shown longer PFS. For instance, Liu et al., 2022, retrospectively evaluated the efficacy and toxicity of fruquintinib in Chinese patients with mCRC and reported median PFS of 5.4 months (95% CI 4.841–5.959) (19). While Song et al., 2021, reported a PFS of 5.1 months (95% CI 3.8–6.4 months) with fruquintinib (10). All these findings suggest towards a significant efficacy of fruquintinib for patients mCRC. The subgroup analysis for PFS in this study also showed the same pattern that revealed fruquintinib had better efficacy as compared to other TKIs in patients receiving two or three previous chemotherapy regimens, those with rectum as primary disease site, with left-sided primary tumor location, with multiple metastasis site and with lung metastasis. In FRESCO study, the subgroup analysis showed that the patients with rectum as primary disease site (HR:0.23; 95%CI: 0.16–0.33) had longer PFS benefit as compared to the patients with colon as primary disease site (HR:0.30; 95%CI: 0.22–0.42), which is aligned with our findings (11). The subgroup analysis results for benefit population of mCRC patients can be used as a reference for further clinical practices.

## 5 Strengths and limitations

To the best of our knowledge, this is the first study comparing fruquintinib trial data with that of an external cohort of RWD using other TKIs. Our study compared the efficacy between TKIs for mCRC in third-line setting using a highly comparative population. PSM was used to adjust for potential confounding factors while selecting the real-world patient’s data for efficacy comparison. The subgroup analyses showed the potentially benefited population with fruquintinib, that could be used as a meaningful reference for clinical practice in future. Also, this ‘Hybrid’ study design is innovative that comprehensively used the trial data for fruquintinib as the RWD is not sufficient, and demonstrated the value of real-world evidence. Due to the nature of ‘Hybrid’ study design, the matched population characteristics are still similar to Phase III trial, and hence the generalizability of these findings is limited. The study by Jin et al., 2021 compared the PFS between regorafenib and fruquintinib in real-world and the findings verified our results with respect to analyses of both overall population and validated population after PSM (20). Nevertheless, due to the RWD is not contained the information of death, effectiveness of fruquintinib in a larger and general real-world population still remains to be investigated. The confounding inherent differences (identified or unidentified) between clinical trial and real-world patients might exist. In addition, since the focus of the present study was to compare the efficacy of fruquintinib with other TKIs, the study has not included other classes of drugs such as TAS-102 which is widely used in the refractory mCRC setting. Thus, a comparison across all third-line treatment options for mCRC is still needed in future research. Further, due to lack of information in RWD, we could neither study genetic mutability such as RAS/BRAF and MSI status nor patient functional status (e.g. ECOG/PS) which is another limitation of the study. Moreover, to minimize the contribution of the differences to the observed outcomes, we tried to mirror inclusion/exclusion criteria of real-world population with FRESCO, using matching method to achieved balance, and following same censoring rules to investigate PFS. However, all the results of the present study should be interpreted with caution since the sample size in few subgroups were limited and baseline characteristics may not all be balanced in all subgroups.

## 6 Conclusion

In summary, with the approach of establishing the external control arm from RWD, this study has demonstrated that treatment with fruquintinib significantly prolonged PFS as compared to other TKIs in patients as third-line mCRC treatment. Our results can be a decent reference for future clinical practices and establish the clinical benefit of fruquintinib for the third-line treatment of mCRC.

## Data availability statement

The original contributions presented in the study are included in the article/**Supplementary Material.**. Further inquiries can be directed to the corresponding author.

## Ethics statement

Ethical review and approval were not required for the study on human participants in accordance with the local legislation and institutional requirements. Written informed consent for participation was not required for this study in accordance with the national legislation and the institutional requirements. This is a study with secondary use of data. At the time of study inclusion, the data had been deidentified to protect subject privacy, and hence the study Consent to Release Information has been exempted.

## Author contributions

YJ conceived and designed the analysis, and collected the data; JL conceived and designed the analysis, and contributed the data; LS conceived and designed the analysis, and contributed the data; JX conceived and designed the analysis, and contributed the data; YZ conceived and designed the analysis, and collected the data; JZ conceived and designed the analysis, and collected the data; HP conceived and designed the analysis, and collected the data; XQ collected the data; YC collected the data; QZ conceived and designed the analysis and wrote the paper; JNL contributed data or analysis tools, performed the analysis and wrote the paper; MS conceived and designed the analysis and wrote the paper; SQ conceived and designed the analysis, contributed the data and wrote the paper. All authors contributed to the article and approved the submitted version.

## Funding

This study was funded by Eli Lilly and Company China Affiliate.

## Acknowledgments

The authors would like to thank Dr Priyanka Bhosale, Anwesha Mandal and Amit Bhat from Indegene Pvt Ltd, India for their medical writing support with inputs from all the authors.

## Conflict of interest

QZ, JNL, and MS were employed by Eli Lilly and Company China Affiliate and hold the share of the company at the time of the study.

The remaining authors declare that the research was conducted in the absence of any commercial or financial relationships that could be construed as a potential conflict of interest.

The authors declare that this study received funding from Eli Lilly and Company China Affiliate. The funder had the following involvement with the study: study design, data collection, analysis, interpretation, and the writing of this report. All authors had full access to all trial data, vouch for the integrity of the data, and had final responsibility for the decision to submit the manuscript for publication.

## Publisher’s note

All claims expressed in this article are solely those of the authors and do not necessarily represent those of their affiliated organizations, or those of the publisher, the editors and the reviewers. Any product that may be evaluated in this article, or claim that may be made by its manufacturer, is not guaranteed or endorsed by the publisher.
