# Supplementary material for: A multi-center effectiveness comparison study of fruquintinib with constructed external control cohort of other targeted kinase inhibitors using real-world data in third-line treatment of metastatic colorectal cancer
Source: Front Oncol. 2022 Nov 24;12:1044328. doi: 10.3389/fonc.2022.1044328 (PMC9730021; doi:10.3389/fonc.2022.1044328)
Supplement: Supplementary file 1 [file Table_1.docx]

Appendix 1: Progression/Censoring Scheme for Analysis of Progression-Free Survival

| No. | Situation | Progression/Censoring Scheme |
| --- | --- | --- |
| 1 | No progression and no death | Censored at date of last evaluable tumor assessment |
| 2 | No baseline tumor assessments  unless they die within two  tumor assessment visits from  randomization | Censored at date of randomization |
| 3 | No on study tumor assessment  unless they die within two  tumor assessment visits from  randomization | Censored at date of randomization |
| 4 | Documented progression other  than situation 6 | Progressed at date of first documented progression per RECIST 1.1 |
| 5 | Death without progression other than situation 6 | Progressed at date of death |
| 6 | Death or progression after two  or more consecutive  missed/non-evaluable tumor  assessments | Censored at date of last evaluable tumor assessment before missed tumor assessments |
| 7 | New anticancer treatment  started prior to documented  disease progression or death on study | Progressed at date of new anticancer treatment, however if  the new anticancer treatment was initiated after two or more consecutive missed/non-evaluable  tumor assessments, subjects will be  censored at date of last evaluable  tumor assessment before missed  tumor assessments |
